# Poly-β-hydroxybutyrate Metabolism Is Unrelated to the Sporulation and Parasporal Crystal Protein Formation in *Bacillus thuringiensis*

**DOI:** 10.3389/fmicb.2016.00836

**Published:** 2016-06-15

**Authors:** Xun Wang, Zhou Li, Xin Li, Hongliang Qian, Xia Cai, Xinfeng Li, Jin He

**Affiliations:** ^1^State Key Laboratory of Agricultural Microbiology, College of Life Science and Technology, Huazhong Agricultural UniversityWuhan, China; ^2^Key Laboratory of Agro-Microbial Resource and Development, Ministry of AgricultureWuhan, China

**Keywords:** poly-3-hydroxybutyrate, *Bacillus thuringiensis*, sporulation, insecticidal crystal proteins (ICPs), PHB synthase (PhaC), depolymerase (PhaZ)

## Abstract

Poly-3-hydroxybutyrate (PHB) is a natural polymer synthesized by many bacteria as a carbon-energy storage material. It was accumulated maximally prior to the spore formation but was degraded during the process of sporulation in *Bacillus thuringiensis*. Intriguingly, *B. thuringiensis* also accumulates large amounts of insecticidal crystal proteins (ICPs) during sporulation, which requires considerable input of carbon and energy sources. How PHB accumulation affects sporulation and ICP formation remains unclear to date. Intuitively, one would imagine that accumulated PHB provides the energy required for ICP formation. Yet our current data indicate that this is not the case. First, growth curves of the deletion mutants of *phaC* (encoding the PHB synthase) and *phaZ* (encoding the PHB depolymerase) were found to be similar to the parent strain BMB171; no difference in growth rate could be observed. In addition we further constructed the *cry1Ac10* ICP gene overexpression strains of BMB171 (BMB171-*cry*), as well as its *phaC* and *phaZ* deletion mutants *ΔphaC*-*cry* and *ΔphaZ*-*cry* to compare their spore and ICP production rates. Again, not much change of ICP production was observed among these strains either. In fact, PHB was still degraded in most *ΔphaZ-cry* cells as observed by transmission electron microscopy. Together these results indicated that there is no direct association between the PHB accumulation and the sporulation and ICP formation in *B. thuringiensis.* Some other enzymes for PHB degradation or other energy source may be responsible for the sporulation and/or ICP formation in *B. thuringiensis*.

## Introduction

*Bacillus thuringiensis* is a ubiquitous Gram-positive, rod-shaped, and spore-forming bacterium that produces poly-3-hydroxybutyrate (PHB) and insecticidal crystal proteins (ICPs). PHB is a linear biopolymer consisting of (R)-3-hydroxybutyrate (3HB) monomers. It can be accumulated as insoluble cytoplasmic granules under over-nutrition state and/or in the absence of one or more essential nutritional elements in *B. thuringiensis* (Jendrossek, 2009). When energy supplies are exhausted, it can then be served as an alternate energy source (Anderson and Dawes, 1990; Jendrossek and Pfeiffer, 2014; Prieto et al., 2016).

The biochemical pathways for PHB synthesis and degradation have been studied in great details in bacteria such as *Ralstonia eutropha* H16, *B. megaterium, Rhodospirillum rubrum*, and so on (Jendrossek and Handrick, 2002). The PHB synthesis requires three enzymatic steps, starting from the condensation of two molecules of acetyl-coenzyme A to form one molecule of acetoacetyl-CoA (condensation reaction, catalyzed by the PhaA thiolase), followed by the reduction of acetoacetyl-CoA to 3-hydroxybutyryl-CoA (3HB-CoA) (reduction reaction, catalyzed by the PhaB reductase), the monomeric precursor for PHB. Finally, the 3HB-CoA monomers are polymerized to form PHB by the PHB synthase (PhaC) (Anderson and Dawes, 1990; Steinbuchel et al., 1992; Madison and Huisman, 1999; Chen, 2009). Likewise, three enzymes of PhaZ, BdhA and AacS are involved for its degradation, in which the most crucial step is the depolymerization of PHB into 3HB-CoA catalyzed by the PHB depolymerase (PhaZ). This enzyme is currently the only known PHB depolymerase observed to date (Tseng et al., 2006; Jendrossek and Pfeiffer, 2014).

The process of sporulation and ICP accumulation requires considerable input of carbon and energy sources (Wang et al., 2013a,b). As early paper (Slepecky and Law, 1961) reported that PHB fueled the sporulation process in *B. megaterium*. It was also reported that PHB was accumulated maximally prior to the spore formation and was degraded during the process of sporulation in *B. cereus* (Kominek and Halvorson, 1965; Valappil et al., 2007). However, how PHB affects sporulation and parasporal crystal formation is still controversial to date. It is generally believed that PHB degradation can provide energy and carbon sources required for the sporulation and parasporal crystal formation. For example, Navarro et al. (2006) found a linear relationship between the PHB accumulation and the parasporal crystal formation in *B. thuringiensis.* Recently, a *phaC* deletion mutant from *B. thuringiensis* BMB 171 was constructed, and found to result in a growth delay and sporulation-deficient phenotype (Chen et al., 2012). However, another group reported that spore formation is not impaired in a *phaC* deletion mutant in *B. thuringiensis* (Chen et al., 2010). In many cases, utilization of PHB does not seem to be imperative for sporulation, because many spore-forming genus *Bacillus* that cannot synthesize PHB still sporulate normally. Most *Bacillus* species such as *B. subtilis* exhibits natural PHB-negative phenotype, and bioimformatic analysis reveals no known *pha* genes or sequences in their genomes (Singh et al., 2009), indicating that PHB has no direct correlation with the bacterial sporulation. McCool and Cannon (2001) constructed a *phaPQRBC* operon deletion strain of *B. megaterium*. However, other than the PHB-negative phenotype, it showed no apparent phenotypic difference and exhibited similar growth rate as its progenitor. Based on these conflicting results, the relationship between PHB accumulation and sporulation and ICP formation still remains obscure. In this study, we have used the markerless gene deletion method, which is believed to cause the least perturbation to genome, to knock-out the *phaC* and *phaZ* genes respectively in order to carefully examine the influence of PHB on sporulation and ICP formation in *B. thuringiensis* BMB171.

## Materials and Methods

### Strains, Plasmids, Primers and Growth Conditions

The strains and plasmids, as well as the primers used in this study, were listed in **Table 1** and Table S1, respectively. *Escherichia coli* were cultured at 37°C in lysogeny broth (LB) medium (g/L: tryptone, 10; yeast extract, 5; NaCl, 10). The medium was adjusted to pH 7.0 before autoclaving at 121°C for 15 min. Unless otherwise specified, *B. thuringiensis* strains were cultured at 28°C in the GYS medium (g/L: glucose, 1.00; yeast extract, 2.00; K_2_HPO_4_⋅3H_2_O, 0.66; (NH_4_)_2_SO_4_, 2.00; MgSO_4_⋅7H_2_O, 0.04; MnSO_4_⋅H_2_O, 0.04; CaCl_2_, 0.08). The medium was autoclaved at 115°C for 30 min after adjusting the pH to 7.8.

**Table 1:** Bacterial strains and plasmids used in this study.

Strains and plasmids	Characteristics	Source
**Strains**		
*E. coli* DH5α	RecA1 endA1 gyrA96 thi hsdR17(r_k_^-^ m_k_^++^) relA1 supE44 Φ80ΔlacZΔM15Δ(lacZYA-argF)U169	Invitrogen
BMB171	*B. thuringiensis* strain BMB171; an acrystalliferous mutant strain; high transformation frequency	Li et al., 2000; He et al., 2010
Δ*phaC*	Markerless *phaC* gene deletion mutant of BMB171	This study
Δ*phaZ*	Markerless *phaZ* gene deletion mutant of BMB171	This study
BMB171*-pHT*	BMB171 strain harboring empty vector pHT304	This study
BMB171*-cry*	BMB171 strain harboring pBMB43-304	This study
Δ*phaC-cry*	Δ*phaC* strain harboring pBMB43-304	This study
Δ*phaZ-cry*	Δ*phaZ* strain harboring pBMB43-304	This study
DH5α-pRP1028	DH5α harboring pRP1028	Janes and Stibitz, 2006
DH5α-pSS4332	DH5α harboring pSS4332	Janes and Stibitz, 2006
DH5α-pSS1827	DH5α harboring pSS1827	Janes and Stibitz, 2006
**Plasmids**		
pSS1827	Helper plasmid for conjugative transfer; Amp^R^	Janes and Stibitz, 2006
pSS4332	*B. thuringiensis*-*E. coli* shuttle plasmid; Km^R^; containing *gfp* and I-*Sce*I restriction enzyme encoding gene	Janes and Stibitz, 2006
pRP1028	*B. thuringiensis*-*E. coli* shuttle plasmid; Amp^R^Erm^R^; containing temperature-sensitive suicide *B. thuringiensis* replicon, *turbo-rfp* gene and anI-*Sce*I recognition site, etc.	Janes and Stibitz, 2006
pRP1161	pRP1028 with upstream homologous arm of *phaC* (*UphaC*) and downstream homologous arm of *phaC* (*DphaC*)	This study
pRP2975	pRP1028 with upstream homologous arm of *phaZ* (*UphaZ* and downstream homologous arm of *phaZ* (*DphaZ*)	This study
pHT304	*B. thuringiensis*–*E. coli* shuttle plasmid; Amp^R^Erm^R^	Arantes and Lereclus, 1991
pBMB43-304	*B. thuringiensis*–*E. coli* shuttle plasmid containing ORF of *cry1Ac10*; Amp^R^Erm^R^	Qi et al., 2015

### Construction of the Integrating Plasmids for Gene Deletion

To construct the plasmids for *phaC* (*BMB171_RS06655*, old locus lag *BMB171_C1161*) and *phaZ* (*BMB171_RS16270*, old locus lag *BMB171_C2975*) deletions, upstream homologous arms of U*phaC* and U*phaD* and downstream homologous arms of D*phaC* and D*phaZ* (homologous to the 5′ and 3′ uncoding regions of the target genes) of approximately 750 bp were amplified from the BMB171 genomic DNA by PCR, using primer pairs of D*phaC*U F/D*phaC*U R, D*phaC*D F/D*phaC*D R, D*phaZ*U F/D*phaZ*U R and D*phaZ*D F/D*phaZ*D R, respectively (Table S1). Then, U*phaC* (or U*phaZ*) was inserted into plasmid pRP1028 between the *Mlu* I and *Bam*H I sites to construct the plasmid pRP1028-U*phaC* (or pRP1028-U*phaZ*). Next, D*phaC* (or D*phaZ*) was inserted into plasmid pRP1028-U*phaC* (or pRP1028-U*phaZ*) between the *Bam*H I and *Kpn* I sites to construct the integrating plasmid pRP1028-UD*phaC* (pRP1161) [or pRP1028-UD*phaZ* (pRP2975)] (Figure S1). pRP1028 was a shuttle plasmid with a temperature-sensitive suicide *B. thuringiensis* replicon. The resulting integrating plasmids were further verified by sequencing.

### Construction of *phaC* and *phaZ* Deletion Strains

The markerless gene deletion system was successfully developed for BMB171 based on an I-*Sce*I mediated replacement method as established in *B. anthracis* by Janes and Stibitz (2006). The detailed procedures have been well described in a previous publication (Zheng et al., 2015), and will only be briefly accounted here taking deletion of *phaC* as the example. Firstly, BMB171 (recipient strain), *E. coli* DH5α containing the integrating plasmid pRP1028-UD*phaC* (donor strain) and *E. coli* DH5α containing the helper plasmid pSS1827 (helper strain) used for conjugational transfer (triparental mating) were grown in LB medium. The plasmid pRP1028-UD*phaC* was integrated into the BMB171 chromosome to form the plasmid-integrated strain by homologous single cross-over recombination. Plasmid-integrated strains were verified by PCR using primer pairs IphaC F/UniversalI R (Table S1). Secondly, the expression plasmid pSS4332 (in DH5α-pSS4332), which produced the restriction endonuclease I-*Sce*I, was conjugationally transfered into the plasmid-integrated strain by a triparental mating system with the help of *E. coli* DH5α-pSS1827 as well. The I-*Sce* I endonuclease can recognize and cleave the highly specific 18 bp DNA target site within the integrated plasmid, resulting in chromosomal double-stranded break and thus stimulated the host genetic repair by homologous recombination between the flanking repeat sequences. As a result, there is approximately 50% possibility to delete the target gene from chromosome. Positive deletion strains of *phaC* were verified by PCR and sequenced (Figures S2 and S3). Finally, the plasmid pSS4332 in the *phaC* deletion strain was eliminated by continuous passage for ten times in the LB medium at 28°C. Those strains in which the pSS4332 plasmid was removed were sensitive against kanamycin. Deletion of *phaZ* was performed using the same method (Figures S2 and S4).

### RNA Extraction, cDNA Synthesis and RT-qPCR

Twenty mililiter of a cultured sample at 48 h in LB medium was collected, and pelleted cells were grounded in liquid nitrogen. The procedures for RNA extraction and cDNA synthesis were carried out as previously described (Wang et al., 2013b; Zheng et al., 2015). Each experiment was repeated in triple.

### Extraction and Determination of ICP Concentrations

Strains of BMB171-*cry*, Δ*phaC*-*cry* and Δ*phaZ*-*cry* were obtained by transformation of the *cry1Ac10* promoter and *cry* gene-containing plasmid (pBMB43-304) (Qi et al., 2015) into the BMB171, Δ*phaC* and Δ*phaZ* strains, respectively. pBMB43-304 is a derivative of the low copy number shuttle plasmid pHT304 (Arantes and Lereclus, 1991), into which a *cry* gene was inserted between the two *Hin*dIII sites. The plasmid pBMB43-304 was maintained by growing the bacteria in the presence of 25 μg/mL erythromycin. For the extraction of the ICP (Cry1Ac10), strains were grown at 28°C and 200 rpm for 20 h in GYS medium supplemented with 25 μg/mL erythromycin. After optical density measurement, cells were diluted to a final OD_600_ of 1.0, and 20 mL of each culture was separately collected by centrifuge at 6000 × *g* for 15 min (AG Eppendorf, Hamburg, Germany). Procedure for the extraction of the ICP was carried out according to a previous study (Wang et al., 2013a). Finally, the ICP was visualized by SDS-PAGE and the ICP concentrations were measured by the Bradford method.

### PHB Assays

The PHB contents of *B. thuringiensis* cells were determined by UV absorption spectroscopy at 245 nm (Law and Slepecky, 1961) using PHB extracted from BMB171 as standard.

### Transmission Electron Microscopy

Sample preparation for transmission electron microscopy was carried out according to the method described in the literature (Tian et al., 2005) with slight modification. Strains were collected at 9 and 19 h, centrifuged at 6000 × *g* for 3 min, and washed three times with PBS buffer (pH 7.2). Pellets were then resuspended in 2.5% glutaraldehyde phosphate buffer, fixed at 4°C for 24 h. After washing three times with PBS buffer (pH 7.2), cells were dehydrated gradually using different concentrations of ethanol (20%, 50%, 70%, 80%, 90%, and 100%). Each process was carried out twice for 15 min each. Samples were stored in vacuum freeze drier overnight. Thin sections were examined on a HITACHI H-7000FA transmission electron microscope (Hitachi, Ibaraki, Japan).

### Virulence Assays

The larvae of *Heliothis armigera* were reared at 28°C with a light/dark cycle of 12:12 h. Artificial diet comprised 4 g yeast extraction, 7 g bean flour, 0.5 g vitamin C, 1.5 g agar, 36% acetic acid and 1 g penicillin per 100 mL of water. The medium was transferred into 24-hole cell culture plates (Corning, USA), 1 mL per well. 200 mL of cells grown in GYS medium for 20 h were harvested by centrifugation (8,500 × *g*, 5 min, 4°C) and suspended in 20 mL distilled water. Cells were then diluted 10,000-fold, with 100 μl of diluted cells applied to each well, allowing it to dry automatically. One first-instar larvae was used for feeding assay per well, with mortality recorded at indicated dates. Meanwhile, body length and weight were measured at the 6th day. Three repeats per bioassay were performed using 24 larvae for each strain (Fiuza et al., 2013).

### Spore Count

Besides BMB171-*cry*, Δ*phaC-cry* and Δ*phaZ-cry* strains, BMB171 and BMB171-*pHT* (BMB171 harboring empty plasmid pHT304) strains were used as controls. OD_600_ of each strain grown in the LB medium at 28°C for 72 h were measured, and diluted to a final OD_600_ of 1.0. Cells were then heated to 65°C for 30 min, followed by gradient dilution (ten times), after that 100 μL of a series of diluents were spreaded onto LB plates. CFU (Colony-forming units) per mL were then counted.

## Results

### PHB Contents Changed upon *phaC* or *phaZ* Deletion

**FIGURE 1:**
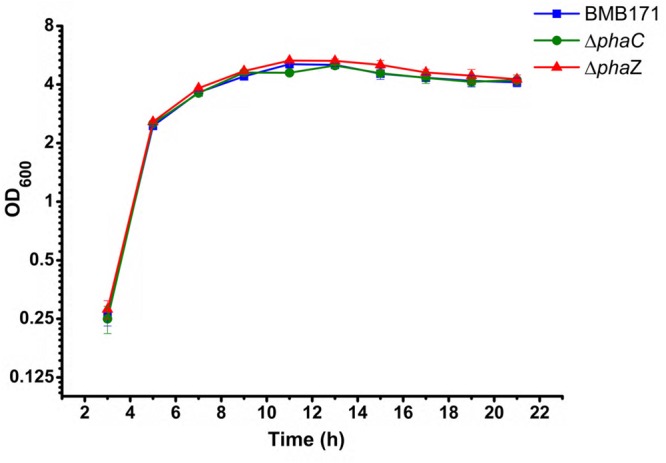
**Growth curves of the BMB171, *ΔphaC* and *ΔphaZ* strains in the GYS medium at 28°C.** A bar for each sampling time point represents standard error of the mean from three batches.

In previous reports, *phaC* and *phaZ* were identified as the genes encoding PHB synthetase and degradation enzyme in *B. thuringiensis* respectively (Tseng et al., 2006; Chen et al., 2010). To disrupt the PHB metabolism pathway, *phaC* and *phaZ* were deleted from the parent strain BMB171 by the markerless gene deletion method, with the corresponding strains named as Δ*phaC* and Δ*phaZ*, respectively. The growth curves of BMB171, Δ*phaC and*Δ*phaZ* strains in GYS medium at 28°C were determined. No obvious difference was observed for all three strains, and they all entered the stationary phase at approximate 11 h (**Figure 1**).

**FIGURE 2:**
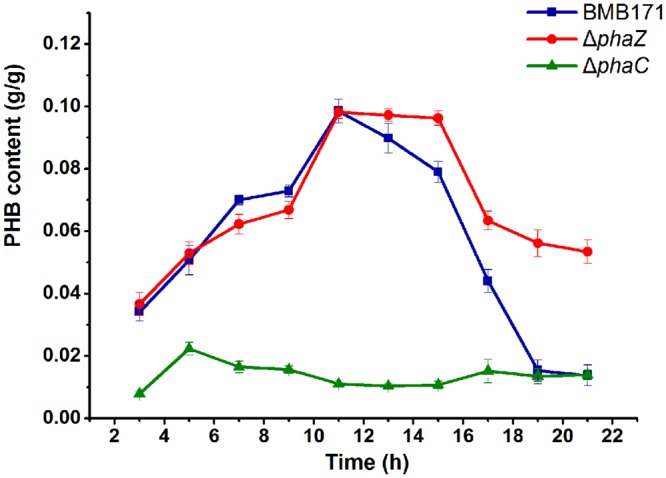
**PHB contents of BMB171, *ΔphaC* and *ΔphaZ* cells.** Cells of indicated strains were grown on the GYS medium at 28°C. Samples were taken at selected time points. Cell dry weight (g/L) and PHB concentration (g/L) were measured, respectively. PHB content (g/g) was calculated by dividing the PHB concentration by the cell dry weight. Each experiment was carried out in triplicate, mean values and standard deviations were calculated.

The amount of PHB produced was measured during the whole growth period. For the parent strain BMB171, it was found that its intracellular PHB level started to accumulate from 3 h and reached to a maximum level at 11 h, after which it descended rapidly to half amount at about 16 h and approximately to zero at 21 h (**Figure 2**). No PHB could be detected in the Δ*phaC* mutant over the whole growth cycle, indicating that PHB accumulation in the Δ*phaC* mutant was totally abolished. This data indicated that PhaC was absolutely required for the PHB synthesis (**Figure 2**). As for the Δ*phaZ* mutant, the accumulation stage was similar, but there was a delay in the degradation stage. It began to degrade at 16 h, with about half amount left at 21 h. Since PHB degradation wasn’t completely abolished in Δ*phaZ*, we speculated that there were some other PHB degradation enzymes or pathways existing in *B. thuringiensis*.

### Cell Morphology Didn’t Change in the *phaC* and *phaZ* Deletion Strains

**FIGURE 3:**
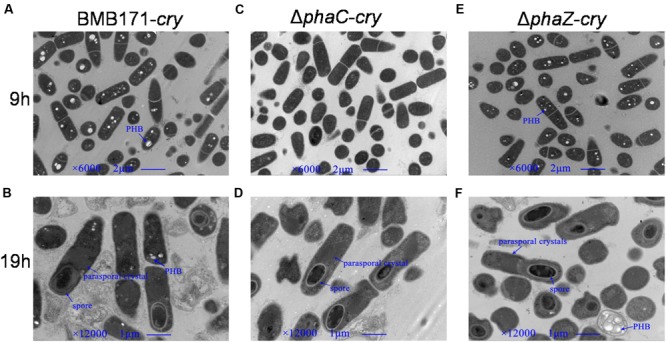
**Transmission electron microscopic observations of PHB (white granule), spore (black ellipse) and parasporal crystal (dark diamond). (A,C)**, and **(E)**, the electron micrographs of BMB171-*cry*, Δ*phaC*-*cry* and Δ*phaZ*-*cry* at 9 h; **(B,D)**, and **(F)**, the corresponding micrographs at 19 h.

BMB171 is an acrystalliferous strain that doesn’t produce ICPs (Li et al., 2000; He et al., 2010). To investigate its morphological changes in the absence of *phaC* and *phaZ*, the ICP coding gene *cry1Ac10*-containing plasmid pBMB43-304 (Qi et al., 2015) needs to be introduced into this strain, and the *ΔphaC* and *ΔphaZ* mutants. The corresponding strains were named as BMB171-*cry, ΔphaC*-*cry* and *ΔphaZ*-*cry*, respectively. Cell morphologies during the exponential phase and sporulation phase were observed using transmission electron microscope. The cell size and shape of the mutants were found to be similar as the parent strain (**Figure 3**). The spore and parasporal crystal morphologies of *ΔphaC*-*cry* and *ΔphaZ*-*cry* also exhibited no major difference compared to those of BMB171-*cry* under the similar growth conditions.

### ICP Formation and Toxicity Were Not Changed in the *ΔphaC-cry* and *ΔphaZ-cry* Strains

**FIGURE 4:**
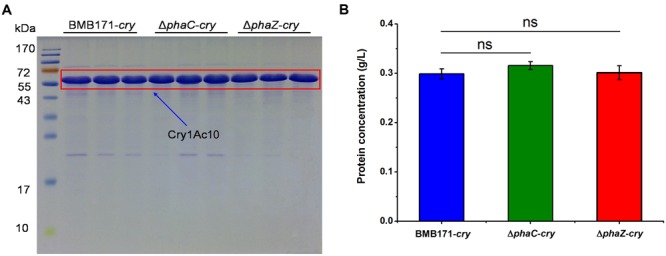
**Effect of *phaC* or *phaZ* deletion on ICP formation. (A)** ICP in BMB171-*cry*, Δ*phaC*-*cry* and Δ*phaZ*-*cry* strains was separated by SDS-PAGE. ICP was boxed in red; **(B)** ICP concentrations measured in BMB171-*cry*, Δ*phaC*-*cry* and Δ*phaZ*-*cry* strains. The values were means ± standard deviations for triplicate assays. Significances of differences by Student’s *t*-test are indicated (ns, *P* > 0.05).

To be more quantitative, we have further measured the ICP amounts to investigate the influence of *phaC* or *phaZ* deletion on ICP formation. As shown in **Figure 4**, all the three strains produce the ICP without conspicuous difference in their amounts (“ns” indicates not significant, *P* > 0.05). It is well known that ICPs are virulent to the *Helicoverpa armigera* larvae. To test whether the toxicities differ in these strains, virulence assays were also performed and the survival rates of the *H. armigera* larvaes fed with these bacteria were measured (H_2_O and BMB171 were set as control groups). **Table 2** shows that the survival rates of BMB171-*cry, ΔphaC*-*cry* and *ΔphaZ*-*cry* were almost similar even after six days (ns, *P* > 0.05). The body length and weight also didn’t differ much among these three strains (ns, *P* > 0.05). Together, these results indicate that the insecticidal activities of the ICP produced by the three strains were comparable.

**Table 2:** Survival rates of *Helicoverpa armigera* larvae fed with BMB171-*cry*, Δ*phaC*-*cry* and Δ*phaZ*-*cry* strains.

Species	Survial rate (%)	Body length (cm)	Weight (mg)
H_2_O	80.6 ± 1.4	1.85 ± 0.10	86.37 ± 10.39
BMB171	78.2 ± 2.9	1.66 ± 0.06	58.57 ± 11.63
BMB171-*cry*	71.8 ± 2.1	1.17 ± 0.09	21.35 ± 3.22
Δ*phaZ-cry*	71.3 ± 2.1 (ns)	1.17 ± 0.09 (ns)	22.80 ± 3.13 (ns)
Δ*phaC-cry*	66.2 ± 2.9 (ns)	1.13 ± 0.04 (ns)	21.35 ± 3.68 (ns)

*The values were means ± standard deviations for triplicate assays. Significances of differences by Student’s *t*-test are indicated that there are no significant differences (ns, *P* > 0.05) on the survival rate, body length and weight of *Helicoverpa armigera* larvae fed with BMB171-*cry*, Δ*phaZ-cry* and Δ*phaC-cry* strains.*

### PhaC or PhaZ is Not Required for Sporulation

**FIGURE 5:**
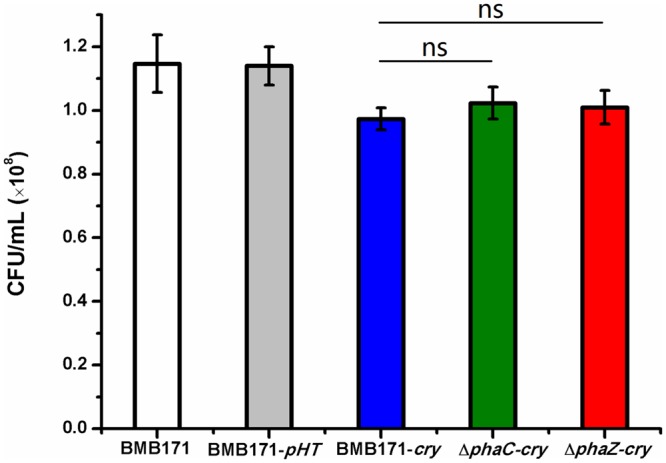
**Spore formation in BMB171, BMB171-*pHT*, BMB171-*cry*, Δ*phaC*-*cry* and Δ*phaZ*-*cry* strains in LB at 72 h.** Spore numbers per mL were indicated by CFU/mL. Each experiment was carried out in triplicate, with mean values and standard deviations calculated. Significances of differences among BMB171-*cry*, Δ*phaC-cry* and Δ*phaZ-cry* by Student’s *t*-test are indicated (ns, *P* > 0.05).

**FIGURE 6:**
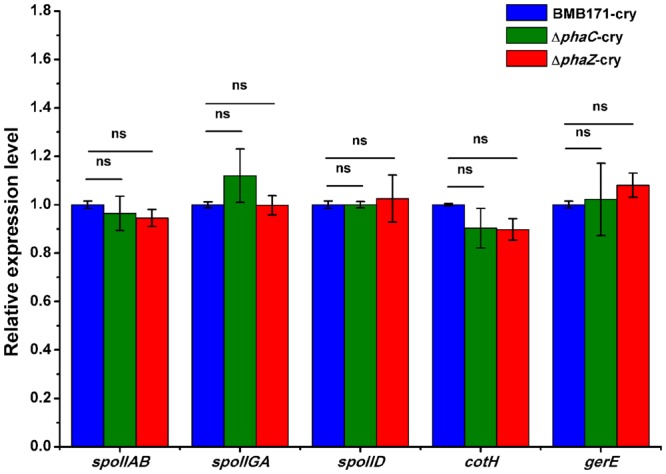
**The relative expression levels of sporulation-related genes in BMB171-*cry*, Δ*phaC*-*cry* and Δ*phaZ*-*cry* strains quantitated by RT-qPCR.** The values were means ± standard deviations for triplicate assays. Significances of differences by Student’s *t*-test are indicated (ns, *P* > 0.05).

As observed by the transmission electron microscopy in the **Figure 3**, both the *ΔphaC-cry* and *ΔphaZ-cry* strains are able to sporulate similar to the BMB171-*cry* strain, which is different to the previous result that sporulation was abolished when *phaC* was deleted from BMB171 (Chen et al., 2012). It is thus important to learn what caused the difference. To make the comparison more objective, three *B. thuringiensis* strains were cultivated in the same condition as Chen’s experiment, and their spore numbers were counted. No significant difference for the BMB171-*cry, ΔphaC*-*cry* and *ΔphaZ*-*cry* strains (**Figure 5**) seems to be present (ns, *P* > 0.05). In addition, several sporulation-related genes were selected to measure their relative expression levels by RT-qPCR experiments using 16S rRNA gene as an internal control. The sporulation-related genes include those expressed in the pre-divisional cells (*spoIIAB* and *spoIIGA*), early mother cells (*spoIID*) and late mother cells (*cotH* and *gerE*) (de Hoon et al., 2010). If the sporulation pathway was impaired in those strains, gene expression level will change. Our data showed that the transcription levels of most sporulation-related genes such as *spoIIAB, spoIIGA, spoIID, cotH* and *gerE* didn’t change significantly (**Figure 6**, ns, *P* > 0.05). Taken together, these results indicate that the spore-forming ability and transcription of sporulation-related genes were not much impaired in the three strains demonstrating that there is no relationship between the PHB accumulation and sporulation in *B. thuringiensis.*

### Bioinformatics Analysis Revealed That There Is No Relationship between the PHB Metabolism and the Sporulation in the Genus *Bacillus*

To further explore the relationship between PHB metabolism and sporulation, we expanded our investigation further through bioinformatics analysis. Since almost all species in the genus *Bacillus* except *B. infantis*can sporulate, we wondered whether those species can also produce PHB. Distribution of *phaC* and *phaZ* genes in the 79 strains of the genus *Bacillus* with complete genomes from NCBI were investigated (Table S2). Screening of metabolic (KEGG) and genomic (NCBI) database for *phaC* and *phaZ* genes revealed that they were absent in the *B. subtilis* group and some other strains in genus *Bacillus* (Table S3). Since PhaC is required for PHB synthesis, we speculate that there is no PHB production in those strains lacking the *phaC* gene. Therefore, no relationship between the PHB accumulation and sporulation could be revealed from this bioinformatics study.

## Discussion

In this study, cell morphologies of BMB171-*cry*, Δ*phaC*-*cry* and Δ*phaZ*-*cry* strains during the exponential and sporulation phases were observed using transmission electron microscope and phase contrast microscope. Interestingly, not much difference in their morphologies were observed. Other features, such as spore production rate and ICP concentration, were also carefully checked. Again, no significant change about these two characteristics was observed among the three strains. These observations were consistent with the data reported by McCool and Cannon (2001) and Chen et al. (2010), in which depletion of PHB metabolism pathway did not affect the sporulation and ICP formation. However, Chen et al. (2012) reported a conflict result even in the same *B. thuringiensis* strain. Comparison of methodologies used in these two experiments revealed that the only difference was the gene knockout method. In our study, we employed a markless gene deletion method to construct the Δ*phaC* and Δ*phaZ* mutant strains, while in Chen’s publication, a traditional knockout method was used, which required a higher screening temperature (42°C) that is close to the lethal temperature (around 45°C) of *B. thuringiensis* (Wu et al., 2011). This temperature difference could lead to a devastating effect on the physiological and genetic properties of this bacterium. Moreover, a resistance gene that was introduced into the chromosome to replace the target gene also increased the possibility of phenotypic change. Thus, we speculated that mutations in other genes other than *phaC* may lead to the lack of sporulation and ICP formation. As a matter of fact, we occasionally observed strains with abnormal phenotype when traditional knockout method was applied. If these unsure knockout strains were chosen for genetic study, inconsistent conclusions could be drawn. Based on our practice in the deletion of hundreds of genes in BMB171, we concluded that this system is easy to operate and allows for clean deletions of one or more genes within an operon (Zheng et al., 2015; Zhang et al., 2015, 2016). Because there is no need for a higher screening temperature, undesirable mutations could decrease, resulting with much more consistent results. Therefore, we strongly suggest using the I-*sec*I endonuclease markless gene knockout method for gene knockout studies in *B. thuringiensis* and even in *B. cereus* group strains.

PHB was generally believed to serve as a sink of carbon and reducing equivalents. However, this doesn’t rule out the possibility that bacteria may use other metabolic pathway to provide the energy required for sporulation and ICP formation. PHB oxidation involves a specific NADH dependent dehydrogenase (PhaB) (Madison and Huisman, 1999), which competes for tricarboxylicacid (TCA) cycle intermediates (for example, NADH, NADPH, ATP, and acetyl-CoA) in the electron transport system. When PHB accumulation is disrupted, more resources are accessible to TCA cycle. Transcriptome analyses of the wild type and mutated *Pseudomonas putida* showed that lack of PHA accumulation in the mutant altered transcription of many genes coding for enzymes involved in the central metabolic pathways, including carbohydrate, fatty acid, amino acid, nucleotide, cofactor and prosthetic group synthesis pathways (Escapa et al., 2012). Meanwhile, when the PHB-negative mutant *R. eutropha* PHB-4 and its wild type were subjected to proteome analyses, higher amounts of acetyl-CoA and pyruvate in the PHB-negative mutant were observed (Raberg et al., 2014). From these results we speculate that when PHB biosynthetic pathway is disrupted, other metabolic pathway could take place to provide the energy required for sporulation and ICP formation (Senior and Dawes, 1973; Jackson and Dawes, 1976; Page and Knosp, 1989; Aldor and Keasling, 2003).

Due to the absence of PHB depolymerase, Δ*phaZ*-*cry* may lost the ability to degrade PHB during stationary phase. However, when *phaZ* was deleted, most bacterial cells can still degrade PHB through an unknown mechanism. Thus deletion of *phaZ* didn’t seem to completely abolish PHB degradation. We speculate that some other pathways may be present to degrade PHB. In fact, many have tried to identify and isolate the PHB depolymerse. PHB is present in two different physical forms, an intracellular native PHB granule form (nPHB) and an extracellular denatured PHB granule form (ePHB). The nPHB exists in an amorphous state and is always coated with various proteins and phospholipase, while the ePHB is a partially crystalline polymer (Jendrossek and Handrick, 2002). The degradation enzymes for nPHB and ePHB displayed a low sequence similarity with rather different biochemical properties (Saegusa et al., 2001; York et al., 2003). Although the ePHB degradation enzyme has been extensively investigated in *P. lemoignei* (Kumar et al., 2000; Schober et al., 2000), not much is known about the nPHB degradation yet. Most methods to identify new nPHB depolymerase is based on sequence comparison with already known depolymerase, either by comparing their *phaZ* genes in the other species, or *phaC* genes (Tseng et al., 2006; Chen et al., 2009; Sznajder and Jendrossek, 2014), or through the blast search of the consensus motif. To date, only one PHB degradation enzyme (PhaZ) was well characterized in *B. thuringiensis* (Tseng et al., 2006; Wang et al., 2014). Obtained results indicated that these methods all suffer from some limitations. Further investigations are required to identify the novel potential nPHB depolymerase. From the results demonstrated in our work, however, we strongly believe that there must have other PHB depolymerases existent in *B. thuringiensis*, and characterization of these potential PHB depolymerases is currently undergoing.

Taken together, we have verified the relationship between PHB accumulation and sporulation and ICP formation in *B. thuringiensis.* This investigation povides a new perspective about the relationship between the important metabolic processes in *B. thuringiensis*, and supply information for designing novel metabolic engineering strategies for maximizing ICPs production or controlling sporulation process.

## Author Contributions

XW performed molecular biology work, participated in study design and drafted the manuscript. ZL performed the phenotype assays and study design. XL (third author) performed the transmission electron microscopy observations, extraction and determination of ICP concentrations, and drafted the manuscript. HQ carried out gene deletions and the virulence assays. XC performed PHB assay and spore counting. XL (sixth author) carried out data analysis. JH conceived the study, participated in the study design, coordinated the research, reviewed and edited the manuscript. All authors read and approved the final manuscript.

## Conflict of Interest Statement

The authors declare that the research was conducted in the absence of any commercial or financial relationships that could be construed as a potential conflict of interest.
