# Supplementary material for: Poly-β-hydroxybutyrate Metabolism Is Unrelated to the Sporulation and Parasporal Crystal Protein Formation in Bacillus thuringiensis
Source: Front Microbiol. 2016 Jun 15;7:836. doi: 10.3389/fmicb.2016.00836 (PMC4908106; doi:10.3389/fmicb.2016.00836)
Supplement: Supplementary file 1 [file Table_1.DOCX]

**Table S1** Primers used in this study

| Primer name | Sequence 5’→3’ |
| --- | --- |
| *DphaCU* F | CGACGCGTCTATAATAGCAGTAAAGAAGCAGC |
| *DphaCU* R | CGCGGATCCCTCCTTTTTGGTCGATTTCCT |
| *DphaCD* F | CGGGATCCCCAACTAGCCTATGTTAGTTGG |
| *DphaCD* R | CGACGTCGACATAATAATATTCTTTGCTAC |
| *DphaZU* F | CCCAAGCTTCATGAAGCATATGATCTCCAACTCC |
| *DphaZU* R | CGACGCGTTCCCCGAAATGAGAAGTGTTG |
| *DphaZD* F | CGACGCGTCACAAGTTCCCCCATTCTATTTAG |
| *DphaZD* R | CGGGATCCCACAGTTGCAACTAAACCAATTTCC |
| *IphaC* F | AGATTCTTTAGGGCAAGCTCCTACA |
| *IphaZ* F | GCTTGCTAAATACGGACCAACGGCA |
| *UniversalI* R | GTGCGAATAAGGGACAGTGAAGAAGAAGG |
| *ISceI* F | GCCATCAAAAAAACCAGGTAATGAACCTGGGTCC |
| *ISceI* R | TTTCAGGAAAGTTTCGGAGGAGATAGTGTTCG |
| *DphaC* F | TATACCTATGCCGTGACGGTGCG |
| *DphaC* R | TGCTTCGCCACTTCCGACTACA |
| *DphaZ* F | AGCTGTTAGTGGCTGCTGCATTT |
| *DphaZ* R | GAAGTCTGGCTGACGATGTGAAA |
| F*phaC-phaB* | CTTCGTCCAAATAACCTCTTTCGGC |
| R*phaB-phaR* | AAAAGAAGAGGTAAAGCCTGCGAAT |
| F*phaQ-phap* | CATTCTTTCCATTTTCTACTTCTCC |
| R*phap-bdhA* | ATTGTTTTTGTAAGGGATGCT |
