# Supplementary material for: Poly-β-hydroxybutyrate Metabolism Is Unrelated to the Sporulation and Parasporal Crystal Protein Formation in Bacillus thuringiensis
Source: Front Microbiol. 2016 Jun 15;7:836. doi: 10.3389/fmicb.2016.00836 (PMC4908106; doi:10.3389/fmicb.2016.00836)
Supplement: Supplementary file 2 [file Table_2.DOCX]

**Table S2.** The distribution of *phaC* and *phaZ* in *Bacillus* genus

| Subgenus | Species | Strains | NC number | *phaC* | *phaZ* |
| --- | --- | --- | --- | --- | --- |
| *B. cereus group* | *B. thuringiensis* | *B. thuringiensis* *serovar konkukian* 97-27 | NC_005957 | *_+_* | *_+_* |
|  |  | *B. thuringiensis* Al Hakam | NC_008600 | *_+_* | *_+_* |
|  |  | *B. thuringiensis* BMB171 | NC_014171 | *_+_* | *_+_* |
|  |  | *B. thuringiensis* *serovar finitimus* YBT-020 | NC_017200 | *_+_* | *_+_* |
|  |  | *B. thuringiensis* *serovar chinensis* CT-43 | NC_017208 | *_+_* | *_+_* |
|  |  | *B. thuringiensis* HD-771 | NC_018500 | *_+_* | *_+_* |
|  |  | [*B. thuringiensis* HD-789](http://www.ncbi.nlm.nih.gov/genome/486?genome_assembly_id=167057) | NC_018508 | *_+_* | *_+_* |
|  |  | *B. thuringiensis* MC28 | NC_018693 | *_+_* | *_+_* |
|  |  | *B. thuringiensis* Bt407 | NC_018877 | *_+_* | *_+_* |
|  |  | *B. thuringiensis serovar kurstaki* HD73 | NC_020238 | *_+_* | *_+_* |
|  |  | *B. thuringiensis* *serovar thuringiensis* IS5056 | NC_020376 | *_+_* | *_+_* |
|  |  | *B. thuringiensis* YBT-1518 | NC_022873 | *_+_* | *_+_* |
|  |  | *B.thuringiensis* *serovar kurstaki* YBT-1520 | CP004858 | *_+_* | *_+_* |
|  | *B.cereus* | *B. cereus* ATCC 14579 | NC_004722 | *_+_* | *_+_* |
|  |  | *B. cereus* ATCC 10987 | NC_003909 | *_+_* | *_+_* |
|  |  | *B. cereus* E33L | NC_006274 | *_+_* | *_+_* |
|  |  | *B. cereus* Q1 | NC_011969 | *_+_* | *_+_* |
|  |  | *B. cereus* B4264 | NC_011725 | *_+_* | *_+_* |
|  |  | *B. cereus* AH187 | NC_011658 | *_+_* | *_+_* |
|  |  | *B. cereus* G9842 | NC_011772 | *_+_* | *_+_* |
|  |  | *B. cereus* AH820 | NC_011773 | *_+_* | *_+_* |
|  |  | *B. cereus* 03BB102 | NC_012472 | *_+_* | *_+_* |
|  |  | *B. cereus* *biovar anthracis* CI | NC_014335 | *_+_* | *_+_* |
|  |  | *B. cereus* F837/76 | NC_016779 | *_+_* | *_+_* |
|  |  | *B. cereus* NC7401 | NC_016771 | *_+_* | *_+_* |
|  |  | *B. cereus* FRI-35 | NC_018491 | *_+_* | *_+_* |
|  | *B.anthracis* | *B. anthracis* Ames | NC_003997 | *_+_* | *_+_* |
|  |  | *B. anthracis* Sterne | NC_005945 | *_+_* | *_+_* |
|  |  | *B. anthracis* 'Ames Ancestor' | NC_007530 | *_+_* | *_+_* |
|  |  | *B. anthracis* CDC 684 | NC_012581 | *_+_* | *_+_* |
|  |  | *B. anthracis* A0248 | NC_012659 | *_+_* | *_+_* |
|  |  | *B. anthracis* H9401 | NC_017729 | *_+_* | *_+_* |
|  |  | *B. anthracis* A16R | CP001974 | *_+_* | *_+_* |
|  |  | *B. anthracis* A16 | CP001970 | *_+_* | *_+_* |
|  | *B.cytotoxicus* | *B. cytotoxicus* NVH 391-98 | NC_009674 | *_+_* | *_+_* |
|  | *B. toyonensis* | *B. toyonensis* BCT-7112 | NC_022781 | *_+_* | *_+_* |
|  | *B. weihenstephanensis* | *B. weihenstephanensis* KBAB4 | NC_010184 | *_+_* | *_+_* |
|  | *B.megaterium* | *B. megaterium* QM B1551 | NC_014019 | *_+_* | *_+_* |
|  |  | *B. megaterium* WSH-002 | NC_017138 | *_+_* | *_+_* |
|  |  | *B. megaterium* DSM319 | NC_014103 | *_+_* | *_+_* |

| Subgenus | Species | Srains | NC number | *phaC* | *phaZ* |
| --- | --- | --- | --- | --- | --- |
| *Bacillus cereus group* | *B.clausii*  *Bacillus thuringiensis* | *B. clausii* KSM-K16 | NC_006582 | *_* | *_* |
|  | *B.coagulans* | *B. coagulans* 2-6 | NC_015634 | *_* | *_* |
|  |  | *B. coagulans* 36D1 | NC_016023 | *_* | *_* |
|  | *B.halodurans* | *B. halodurans* C-125 | NC_022524 | *_* | *_* |
|  | *B. infantis* | *B. infantis* NRRL B-14911 | NC_022524 | *_* | *_* |
|  | *B.pseudofirmus* | *B. pseudofirmus* OF4 | NC_013791 | *_* | *_* |
|  | *B. pumilus* | *B. pumilus* SAFR-032 | NC_009848 | *_* | *_* |
| *B. subtilis group* | *B.amyloliquefaciens* | *B. amyloliquefaciens* DSM 7 | NC_014551 | *_* | *_* |
|  |  | *B.amyloliquefaciens subsp. plantarum* FZB42 | NC_009725 | *_* | *_* |
|  |  | *B. amyloliquefaciens* IT-45 | NC_020272 | *_* | *_* |
|  |  | *B. amyloliquefaciens* LFB112 | NC_023073 | *_* | *_* |
|  |  | *B. amyloliquefaciens* LL3 | NC_017190 | *_* | *_* |
|  |  | *B. amyloliquefaciens subsp. plantarum* AS43.3 | NC_019842 | *_* | *_* |
|  |  | *B. amyloliquefaciens subsp. plantarum* CAU B946 | NC_016784 | *_* | *_* |
|  |  | *B. amyloliquefaciens subsp. plantarum* NAU-B3 | NC_022530 | *_* | *_* |
|  |  | *B. amyloliquefaciens subsp. plantarum* UCMB5033 | NC_022075 | *_* | *_* |
|  |  | *B. amyloliquefaciens subsp. plantarum* UCMB5036 | NC_020410 | *_* | *_* |
|  |  | *B. amyloliquefaciens subsp. plantarum* UCMB5113 | NC_022081 | *_* | *_* |
|  |  | *B. amyloliquefaciens subsp. plantarum* YAU B9601-Y2 | NC_017061 | *_* | *_* |
|  |  | *B. amyloliquefaciens* TA208 | NC_017188 | *_* | *_* |
|  |  | *B. amyloliquefaciens* XH7 | NC_017191 | *_* | *_* |
|  |  | *B. amyloliquefaciens* Y2 | NC_017912 | *_* | *_* |
|  | *B.atrophaeus* | *B. atrophaeus* 1942 | NC_014639 | *_* | *_* |
|  | *B.licheniformis* | *B. licheniformis* 9945A | NC_021362 | *_* | *_* |
|  |  | *B. licheniformis* DSM 13 = ATCC 14580 | NC_006322 | *_* | *_* |
|  |  | *B. licheniformis* ATCC 14580 | NC_006270 | *_* | *_* |
|  | *B. subtilis* | *B. subtilis subsp. subtilis* 168 | NC_000964 | *_* | *_* |
|  |  | *B. subtilis subsp. subtilis* 6051-HGW | NC_020507 | *_* | *_* |
|  |  | *B. subtilis subsp. subtilis str*. BAB-1 | NC_020832 | *_* | *_* |
|  |  | *B. subtilis* BSn5 | NC_014976 | *_* | *_* |
|  |  | *B. subtilis subsp. subtilis* BSP1 | NC_019896 | *_* | *_* |
|  |  | *B. subtilis subsp*. natto BEST195 | NC_017196 | *_* | *_* |
|  |  | *B. subtilis* PY79 | NC_022898 | *_* | *_* |
|  |  | *B. subtilis* QB928 | NC_018520 | *_* | *_* |
|  |  | *B. subtilis subsp. subtilis str.* RO-NN-1 | NC_017195 | *_* | *_* |
|  |  | *B. subtilis subsp. spizizenii* W23 | NC_014479 | *_* | *_* |
|  |  | *B. subtilis* XF-1 | NC_020244 | *_* | *_* |
|  |  | *Bacillus sp*. 1NLA3E | NC_021171 | *_* | *_* |
|  |  | *Bacillus sp*. JS | NC_017743 | *_* | *_* |
