# Supplementary material for: Poly-β-hydroxybutyrate Metabolism Is Unrelated to the Sporulation and Parasporal Crystal Protein Formation in Bacillus thuringiensis
Source: Front Microbiol. 2016 Jun 15;7:836. doi: 10.3389/fmicb.2016.00836 (PMC4908106; doi:10.3389/fmicb.2016.00836)
Supplement: Supplementary file 3 [file Table_3.DOCX]

**Table S3**. The distribution of *phaC* and *phaZ* gene and the spore-forming ability in genus *Bacillus*.

| Subgenus | Species | *phaC* | *phaZ* | Spore-forming |
| --- | --- | --- | --- | --- |
| *B. cereus group* | *B. thuringiensis* | + | + | + |
|  | *B.cereus* | + | + | + |
|  | *B.anthracis* | + | + | + |
|  | *B.cytotoxicus* | + | + | + |
|  | *B. toyonensis* | + | + | + |
|  | *B. weihenstephanensis* | + | + | + |
|  | *B.megaterium* | + | + | + |
|  | *B.clausii* | _ | _ | + |
|  | *B.coagulans* | _ | _ | + |
|  | *B.halodurans* | _ | _ | + |
|  | *B. infantis* | _ | _ | _ |
|  | *B.pseudofirmus* | _ | _ | + |
|  | *B. pumilus* | _ | _ | + |
| *B. subtilis group* | *B.amyloliquefaciens* | _ | _ | + |
|  | *B.atrophaeus* | _ | _ | + |
|  | *B.licheniformis* | _ | _ | + |
|  | *B. subtilis* | _ | _ | + |
