# Supplementary material for: Poly-β-hydroxybutyrate Metabolism Is Unrelated to the Sporulation and Parasporal Crystal Protein Formation in Bacillus thuringiensis
Source: Front Microbiol. 2016 Jun 15;7:836. doi: 10.3389/fmicb.2016.00836 (PMC4908106; doi:10.3389/fmicb.2016.00836)
Supplement: Supplementary file 4 [file Presentation_1.PDF]

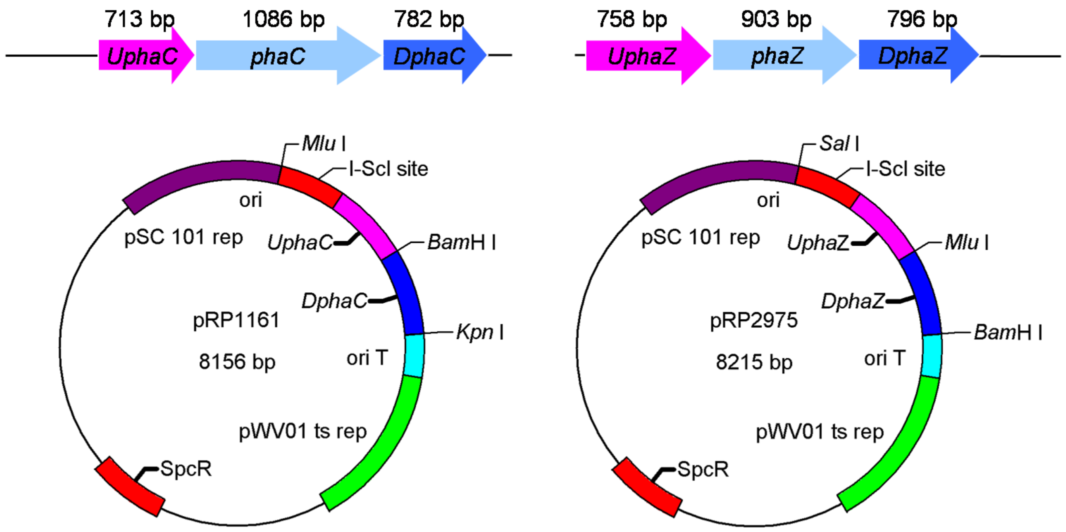


**Figure S1. Integrating plasmids pRP1161 and pRP2975 constructed for the *phaC* and *phaZ* gene deletion, respectively.** The upstream and downstream homologous arms of *phaC* or *phaZ* were cloned into vector indicated in pink and blue between the cloning sites *Mlu*I and *Bam*HI, and *BamH*I and *Kpn*I.
