# Supplementary material for: Poly-β-hydroxybutyrate Metabolism Is Unrelated to the Sporulation and Parasporal Crystal Protein Formation in Bacillus thuringiensis
Source: Front Microbiol. 2016 Jun 15;7:836. doi: 10.3389/fmicb.2016.00836 (PMC4908106; doi:10.3389/fmicb.2016.00836)
Supplement: Supplementary file 5 [file Presentation_2.PDF]

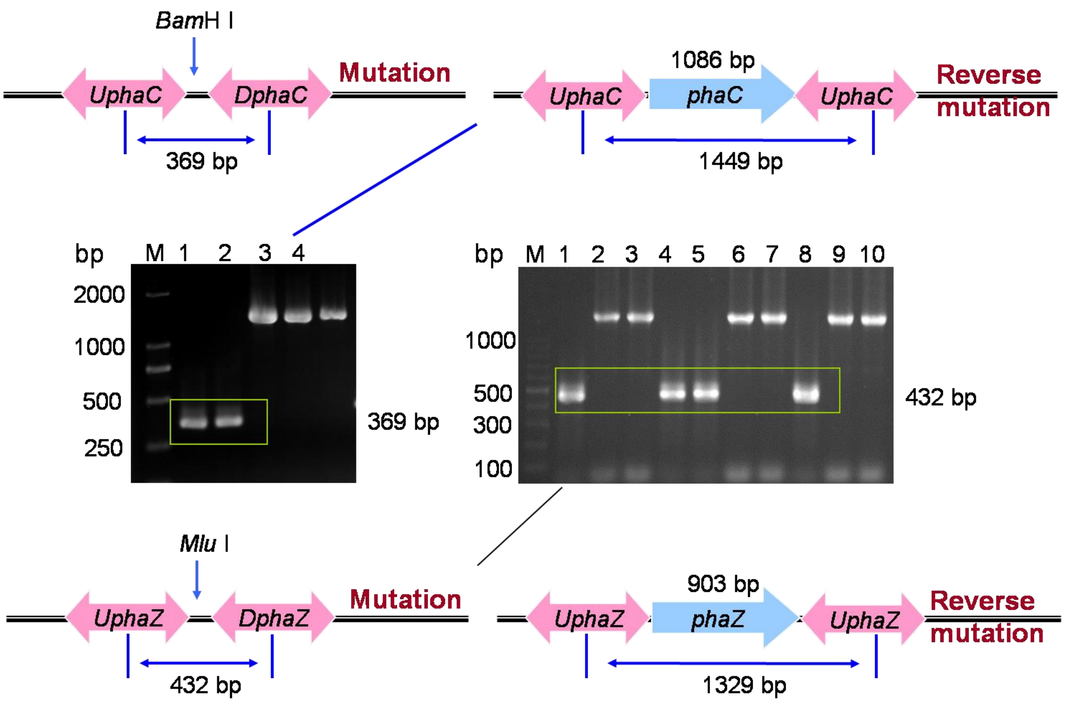

Figure S2. Verification of *phaC* and *phaZ* gene deletion by PCR. PCR products were separated using 1% agarose gel electrophoresis. The PCR primers were indicated as blue vertical bar. The left DNA image is the PCR result for screening *phaC* deletion strain. The PCR product size of *phaC* deleted strain is 368 bp, while WT is 1449 bp. Lanes 1 and 2 are successful *phaC*-deleted strain. The right DNA image is the PCR result for screening *phaC* deletion strain. Lanes 1, 4, 5, 8 are successful *phaZ*-deleted strain.
