# Supplementary material for: Poly-β-hydroxybutyrate Metabolism Is Unrelated to the Sporulation and Parasporal Crystal Protein Formation in Bacillus thuringiensis
Source: Front Microbiol. 2016 Jun 15;7:836. doi: 10.3389/fmicb.2016.00836 (PMC4908106; doi:10.3389/fmicb.2016.00836)
Supplement: Supplementary file 6 [file Presentation_3.PDF]

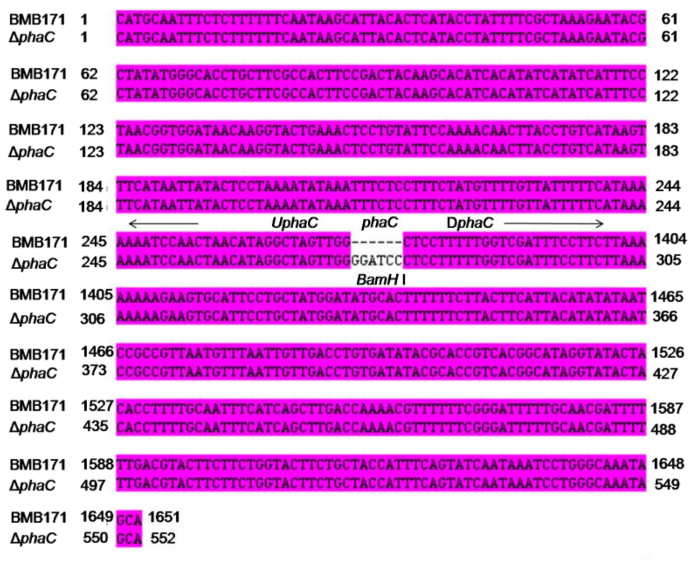

Figure S3. Verification of *phaC* deletion by sequencing. Sequence alignment of PCR products amplified from the △*phaC* genomic DNA and the BMB171 genomic DNA using primer pair *DphaC* F /*DphaC* R. The restriction site of *BamH* I GGATCC residues in the *phaC* locus of the BMB171 chromosome (NC_014171, GI: 296500838). Missing sequence of gene *phaC* also listed as follows:

ATGactacattcgcaacagaatgggaaaagcaattagagctatacccagaagagtaccgaaaagcataccgccgagtgaaaagggcgagtgaa
attttattgcgtgaaccagagccgcaagtcggattaacgccgaaagaggttatttggacgaagaataagacgaagctttatcgctacattccaaaacaa
gaaaaaacacaaagagttccaattctgttaatatatgctcttattaataaaccatatattatggatttaactcctggaaatagtttagtggaatatctagtggat
cgtggttttgatgtatatatgcttgattggggcacatttggtttagaagatagtcatttgaaatttgatgatttcgtgtttgattatattgcaaaagcagtaaaaa
aagtaatgcgaactgcaaaatcggacgagatttctttacttggttattgcatgggtggaacgctaacttctatttatgcagcgcttcatccgcacatgccaa
ttcgtaatttaattttcatgacaagtccttttgatttctctgaaacaggattgtatggtcctttattagatgagaaatacttcaatttagataaagcggttgataca
tttggaaatattccgccagaaatgattgatttcggaaacaaaatgttaaagccaattacgaactttgttggtccatatgttgctctagtagatcgttcagaga
atgaacgcttcgtcgaaagctggagattggttcaaaagtgggttggtgatggtattccgttcccaggtgaatcatacagacagtggattcgtgatttttat
caaaataataaattggttaagggtgaactcgttattcgcggacaaaaggtagatcttgcaaatattaaggcgaatgtcttaaatatttccgggaaacgtga
tcatatcgctttgccatgtcaagtagaagcattactagaccatatttctagcacagataaacaatatgtatgtttaccaacagggcatatgtctatcgtttac
ggtggaacagctgtaaaacaaacatatccgacgattggaaattggcttgaagagcgttctaatTAA
