# Supplementary material for: Poly-β-hydroxybutyrate Metabolism Is Unrelated to the Sporulation and Parasporal Crystal Protein Formation in Bacillus thuringiensis
Source: Front Microbiol. 2016 Jun 15;7:836. doi: 10.3389/fmicb.2016.00836 (PMC4908106; doi:10.3389/fmicb.2016.00836)
Supplement: Supplementary file 7 [file Presentation_4.PDF]

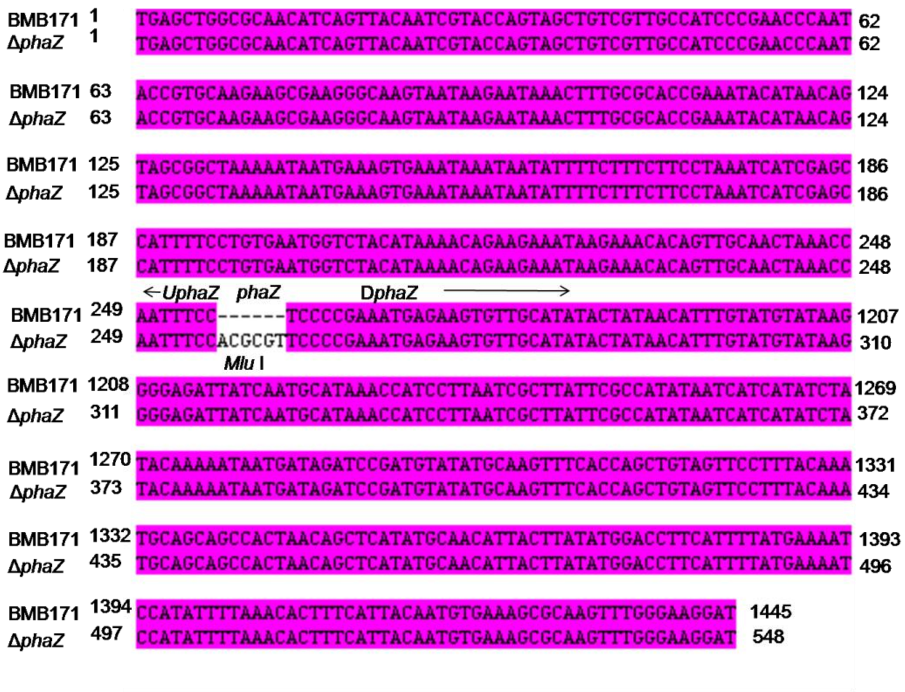

Figure S4. Verification of △*phaZ* by sequencing. Sequence alignment of PCR products amplified from the △*phaZ* genomic DNA and the BMB171 genomic DNA using primer pair *DphaZ* F /*DphaZ* R. The restriction site of *Mlu* I ACGCGT residues in the *phaZ* locus of the BMB171 chromosome (NC_014171, GI: 296500838). Missing sequence of gene *phaZ* also listed as follows:

ATGattaagcctgcaacaatggagtttgtttcactatcgaacggagaaacgattgcatatcaggaagttggaaggcgaaatacagatattcttgtact
cattcacgggaacatgacatcgtcacaacattgggatttagttattgaaaagttgcaagatcaataccatatttacgctcttgatttaagagggtttggaca
atcaacatataatcagtcgatagattcattacaagactttgcagacgatgtaaaattatttattgacgagttaaagctagagaaattttcattaatgggctgg
tcaatgggtggtggcgttgcgatgcaatttacagcgaatcacccaacttttgtagaaaagttaattttagtagaatcagtaggaatgaaaggatacccaat
ctttaaaaaagatacgaatgggcagccgattgtatcaagtttagtaaagacgaaagaagaaattgcgcaagatccagtacaaatcgctccagtattaga
tgcgataaaaaatatgaacaaactatattaccgtacagtatggaatctattaatatatacacataatcaacctgaaccggatcgttatgaaaagtatttagat
gatatgttaacgcaacgtaatttcgtagatgtgaattatgcgctcattacatttaatatttcagatgaacataatggggttgtagagggaagtaaacaaattc
atcgtattaaagcgccaacactcgtcatacaaggtgatagagattatgtcgtaccgcaagtagtcggtgaggaattagcgaaacatttgccaaatgcag
agttgaaggtattagaagattgcggacactcgccgtttattgattgtttagatgtatttataaaacatgtagaggattggttagaacagaaaTAA
